# Thymoma-Associated Paraneoplastic Autoimmune Multiorgan Syndrome—From Pemphigus to Lichenoid Dermatitis

**DOI:** 10.3389/fimmu.2019.01413

**Published:** 2019-06-21

**Authors:** Farzan Solimani, Roberto Maglie, Robert Pollmann, Thomas Schmidt, Ansgar Schmidt, Norito Ishii, Björn Tackenberg, Andreas Kirschbaum, Dario Didona, Julia Pickert, Rüdiger Eming, Takashi Hashimoto, Michael Hertl

**Affiliations:** ^1^Department of Dermatology and Allergology, Philipps-University, Marburg, Germany; ^2^Institute of Pathology, Philipps-University, Marburg, Germany; ^3^Department of Dermatology, Kurume University School of Medicine, and Kurume University Institute of Cutaneous Cell Biology, Kurume, Japan; ^4^Department of Neuroimmunology, Philipps-University, Marburg, Germany; ^5^Department of Surgery, Philipps-University, Marburg, Germany; ^6^Osaka City University Graduate School of Medicine, Osaka, Japan

**Keywords:** *Pemphigus foliaceous*, autoimmunity, thymoma auto-immunity, PAMS, myasthenia (myasthenia gravis—MG), GVHD-like disease

## Abstract

**Introduction:** Paraneoplastic autoimmune multi-organ syndrome (PAMS) is a rare clinical condition characterized by variable and heterogeneous clinical phenotypes in the presence of neoplasias which largely depend on the activation of humoral and cellular immune responses. Clinically, these patients present with a spectrum of antibody-driven pemphigus-like lesions to graft-vs.-host-disease-like exanthemas with a lichenoid inflammatory infiltrate in the skin. PAMS is occasionally associated with thymoma, in which altered immune surveillance eventually leads to multiorgan autoimmunity which often includes variable cutaneous symptoms. This disorder is associated with a profound disturbance of peripheral immune tolerance against human autoantigens.

**Objectives:** We here present a patient with relapsing thymoma who developed PAMS with several cutaneous and extracutaneous autoimmune disorders.

**Materials:** Peripheral blood mononuclear cells (PBMC), sera, and lesional skin biopsies were obtained at different clinical disease stages. Peripheral T cell subsets were characterized phenotypically and the cytokine profile of the peripheral blood T cellular response against distinct epidermal and dermal autoantigens of the skin was analyzed by ELISpot assay. Serological screening was performed by ELISA and immunoblot analysis. Skin biopsies were subjected to immunohistochemical analysis of distinct T cell subsets. Thymoma tissue was analyzed for the presence of T regulatory cells and compared with adult thymus and indolent thymoma.

**Results and Conclusions:** In the present case, thymoma was the cause of the observed multi-organ autoimmune syndromes as its recurrence and surgical removal was associated with the relapse and regression of the cutaneous symptoms, respectively. Initially, the patient presented with two autoimmune disorders with Th2/Th1 imbalance, myasthenia gravis (MG) and pemphigus foliaceus (PF), which regressed upon immunosuppressive treatment. Months later, the patient developed a lichenoid exanthema with a Th1-dominated skin infiltrate. Further clinical evaluation revealed the recurrence of the thymoma and the lichenoid exanthema gradually regressed upon thymectomy. Our contention that T cell recognition against distinct cutaneous autoantigens, such as desmoglein 1 (Dsg1), shifted from a Th2 to a Th1-dominated immune response could not be fully substantiated as the patient was on a stringent immunosuppressive treatment regimen. We could only observe a decrease of the initially present serum IgG autoantibodies against Dsg1. Phenotypic analysis of the associated thymoma showed a lower number of T regulatory cells compared to adult thymus and indolent thymoma, suggesting that impaired thymus-derived immune surveillance had a direct impact on the outcome of the observed cutaneous autoimmune disorders.

## Background

PAMS is an extremely rare clinical syndrome which arises in patients with lymphoproliferative or solid tumors, like thymomas (1, 2). It is increasingly debated whether PAMS and paraneoplastic pemphigus (PNP) should be considered as one entity, since, in both cases, production of IgG autoantibodies against desmosomal adhesions molecules, such as Dsg1 and/or Dsg3, are related to an altered immune surveillance induced by the underlying neoplasia (3–5). In contrast to pemphigus vulgaris (PV) or PF, two well-characterized autoimmune bullous disorders of the skin associated with IgG autoantibodies against Dsg3 and Dsg1, respectively, PAMS is characterized by a wide clinical heterogeneity (6), ranging from classic pemphigus-like muco-cutaneous lesions with erosions, and blisters to lichenoid, graft vs. host disease (GVHD)-like, bullous pemphigoid (BP)-like, and erythema multiforme like-skin lesions (1). As in PNP, mortality of PAMS is high and largely dependent on the underlying malignancy or opportunistic infections (6, 7). As previously reported, PAMS may be associated with thymoma, a neoplasm which arises from epithelial cells of the thymus and accounts for ~50% of all mediastinal tumors (8–15).

Thymomas are often asymptomatic and are sometimes detected by chance by routine radiographic examinations (16). Nonetheless, given the central role of the thymus in adaptive immune regulation, ensuring T-cell tolerance against self-antigens and preventing the maturation of self-reactive T-cells, it is not surprising that thymomas can frequently lead to autoimmune syndromes (7, 17). Approximately 50% of the patients with thymomas experience associated autoimmune diseases, including MG, pure red cell aplasia, systemic lupus erythematosus, and Goodpasture's syndrome (17, 18). Cutaneous disorders associated with thymomas are widely heterogeneous and include pemphigus, BP, lichen planus (LP), vitiligo, alopecia areata, and lupus erythematosus (17).

## Case Presentation

**Figure 1:**
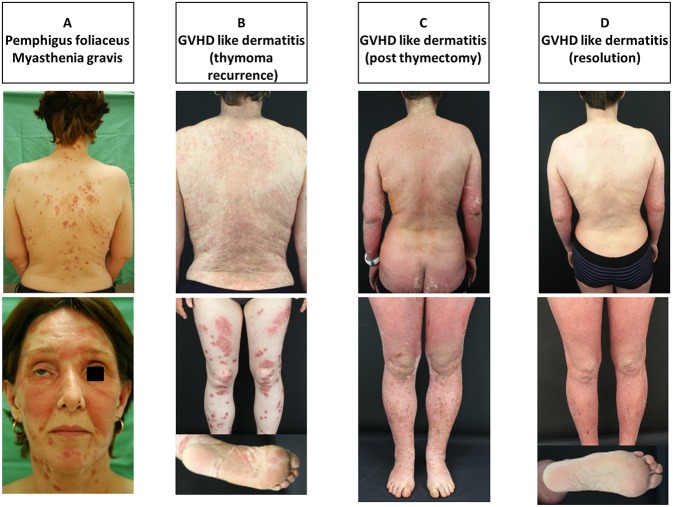
**(A–D)** Clinical manifestations of the patient during clinical observational period. **(A)** Initially pemphigus foliaceus with involvement of the seborrheic skin areas and ptosis in myasthenia gravis, **(B)** lichenoid eruption at the time of thymoma recurrence, **(C)** erythroderma with lichenoid eruption right after thymectomy, and **(D)** resolution of the lichenoid eruption.

A 51 year old Caucasian woman presented with a diffuse skin rash associated with abdominal pain and diarrhea with a 2 weeks duration. Her past medical history was remarkable for a B2-type thymoma which was diagnosed about 7 years earlier. The patient underwent complete surgical resection of the tumor and then received adjuvant radiotherapy (50.4 Gy). Few months after surgical removal of the thymoma, the patient developed diffuse muscle weakness and was diagnosed with MG. She was started on azathioprine and pyridostigmine resulting in a good clinical control of her symptoms. Physical examination revealed erythematous plaques with shallow erosions and overwhelming yellow-to-brown crusts, involving the trunk, mainly back, upper and, to lesser extent, lower limbs, dorsal aspect of hands, face, and scalp (Figure 1A). There was no mucosal involvement. The results of the routine laboratory investigations were unremarkable except for elevated serum concentrations of the transaminases, GOT and GPT.

**Figure 2:**
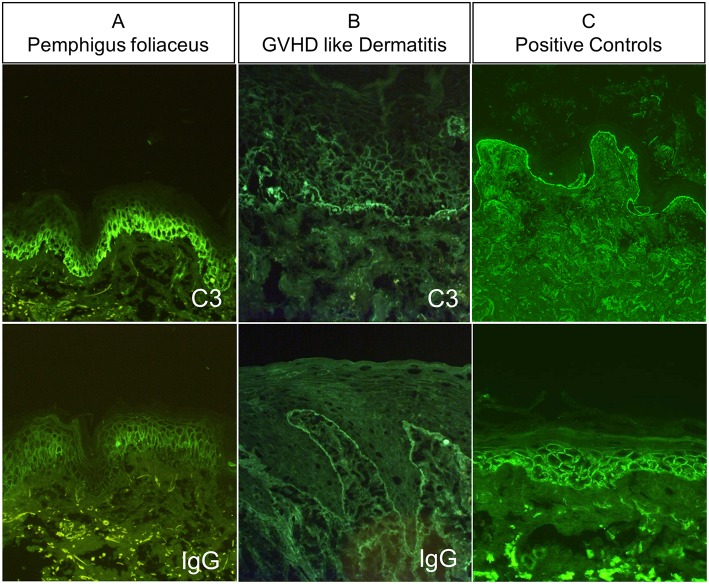
Immune serological characteristics of the patient, **(A)** direct immunofluorescence (DIF) at time of pemphigus foliaceus, which showed IgG and C3 deposits at the surface of epidermal keratinocytes and **(B)** DIF at time of GVHD-like dermatitis which showed IgG C3 deposits along the BMZ. **(C)** positive DIF controls of perilesional BP skin, showing linear deposition of IgG along the BMZ (above), and positive DIF from perilesional PV skin presenting deposits at the surface of epidermal keratinocytes.

Initially, after surgical removal of the thymoma, direct immunofluorescence (DIF) from perilesional biopsy of the scaly erythematous skin rash revealed deposits of both IgG and C3 on the surface of epidermal keratinocytes (Figures 2A,C). Another DIF which was taken later at the time of GVHD-like dermatitis revealed instead linear deposits of IgG and C3 along the dermal-epidermal basement membrane zone (BMZ) (Figures 2B,C). Indirect immunofluorescence (IIF) on normal human skin, 1M NaCl-split human skin and monkey esophagus showed neither IgG autoantibodies to either the surface of epithelial cells nor on the dermal-epidermal BMZ (not shown). In addition, IIF on rat bladder was negative on transitional epithelia (not shown). Of note, the patient had IgG autoantibodies against Dsg1 (719 relative units (RU)/ml, cutoff <20 RE/ml) and desmocollin 1 (Dsc1) (0.448 OD, cutoff <0.200 OD) by ELISA and IgG autoantibodies against laminin 332 by immunoblot analysis (Table 1).

**Table 1:** Autoantibody profile of the studied patient with thymoma-associated paraneoplastic autoimmune multiorgan syndrome.

**Antigens**	**pemphigus foliaceus + myasthenia gravis**	**Thymoma reoccurrence**	**After thymectomy**	**Immunoassay**
Dsg1	719 RE/ml (Cutoff <20 RU/ml)	71 RU/ml	6 RU/ml	ELISA
Dsg3	0 (Cutoff <20 RU/ml)	0	0	ELISA
Desmocollin 1	0.448 OD (Cutoff <0.200 OD)	nd	nd	ELISA
Plakins	–	–	–	Immunoblot
BP180	0 (Cutoff <20 RU/ml)	0	0	ELISA
BP230	0 (Cutoff <20 RU/ml)	0	0	ELISA
Laminin γ-1	–	–	–	Immunoblot
Laminin 332	+	+	+	Immunoblot
Human type VII collagen	0 (Cutoff <6 RU/ml)	0	0	ELISA
Skeletal muscle (titin)	++	+	–	BIOCHIP MOSAIC
Parotid gland	–	nd	nd	BIOCHIP MOSAIC
Stomach and bowels	–	nd	nd	BIOCHIP MOSAIC
Granulocytes, eosinophils, platelets, lymphocytes	–	nd	nd	BIOCHIP MOSAIC
Spinal cord, cerebrum, nerves, and cerebellum	–	nd	nd	BIOCHIP MOSAIC
HEP-2 cells	–	nd	nd	BIOCHIP MOSAIC
Liver, kidney, heart	–	nd	nd	BIOCHIP MOSAIC

*nd, not determined; OD, optical densitiy; RU, relative units*.

Based on clinical, histologic, and immunologic findings, the diagnosis of PF with additional anti-BMZ IgG reactivity was established. As there was neither evidence for IgG antibodies against desmosomal plaque proteins nor the 170 kDa alpha2-macroglobulin-like protein-1, the diagnosis of PNP was abandoned.

The patient initially received a cycle of intravenous immunoglobulins (IVIg) at 2 g/kg/cycle, followed by infusions of rituximab 2 × 1 g two weeks apart (19, 20). The patient's skin erosions improved significantly and fully regressed eventually, in association with the decrease of anti-Dsg1 serum IgG antibodies.

**Figure 3:**
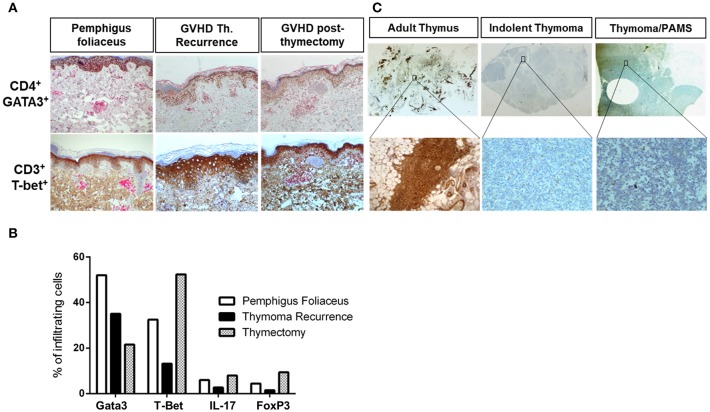
**(A)** Immunohistochemical analysis of T cell subsets in pemphigus foliaceus (PF) and lichenoid skin lesions during the observational period. **(A)** CD3^+^/Tbet^+^ and CD4^+^/GATA3^+^ T cell skin infiltrate in PF, the lichenoid eruption at thymoma recurrence, and after thymectomy. **(B)** Percentage of CD4^+^/GATA3^+^, T-Bet+, IL17A^+^, and FoxP3^+^ T cells in PF skin lesions, at thymoma recurrence and after thymectomy, **(C)** expression pattern of FoxP3+ T cells in adult thymus, indolent thymoma, and in PAMS-associated thymoma (present case).

Several months later, she developed diffuse erythroderma (Figure 1B). Erythematous targetoid plaques, resembling erythema multiforme (EM), and hyperkeratotic plaques appeared at her lower limbs and soles, respectively (Figure 1B). A skin biopsy from the erythrodermic skin revealed liquefactive degeneration and apoptotic keratinocytes and a band-like lymphocytic infiltrate along the BMZ (Figure 3A). These cutaneous symptoms were associated with persistent diarrhea and elevated liver enzymes and were thus considered as thymoma-associated GVHD-like disease. A chest X-ray and magnetic resonance imaging revealed a large mass in the left anterior mediastinum which was diagnosed by histopathology as a recurrent type-B2 thymoma. Prior to surgical removal, the patient received a cycle of intravenous cyclophosphamide (1,300 mg total dose) and IVIg (2 g/kg given on three consecutive days). After tumor resection, erythroderma with multiform lesions gradually regressed (Figure 1C) and eventually disappeared (Figure 1D). Of note, MG significantly improved and anti-Dsg1 serum IgG antibodies were no longer detectable (Table 1). A third skin biopsy revealed findings consistent with GVHD-like erythroderma (Figure 3A). Despite the clinical response, the patient eventually died because of an opportunistic bacterial infection leading to fulminant sepsis.

## Laboratory Investigations

### Autoantibody Profile in the Patient With Thymoma-Associated Autoimmune Syndrome

The patient's serum IgG antibodies were reactive with Dsg1 and Dsc1 by ELISA and laminin 332 by immunoblot analysis (Table 1). Immunoblot analysis with epidermal extracts failed to identify IgG against plakins including periplakin and envoplakin (Table 1). Autoantibody profiling against various tissues showed the presence of IgG antibodies against muscle tissue (anti-titin IgG antibodies) which is characteristic for MG (Table 1) (21). More detailed information regarding the IgG autoantibody profile is given in the Supplementary Material. For information regarding detection of autoantibodies against laminin-γ1 please see (22).

### Immunohistochemical Analysis of the T Cell Infiltrate in Skin Lesions and Thymoma Tissue

Skin samples taken from PF lesions, pre- and post-thymectomy GVHD-like skin lesions were further analyzed by immunohistochemistry as recently described (23) (more detailed information is given in the Supplementary Materials). PF skin lesions showed more CD4^+^GATA-3^+^ Th2 cells than CD3^+^T-Bet^+^ compared to the Th1-dominated GVHD-like skin lesions which inversely showed a stronger presence of CD3^+^T-Bet^+^ Th1 cells than CD4^+^GATA-3^+^ Th2 cells (Figures 3A,B). The numbers of IL-17^+^ T cells and of FoxP3^+^ Treg cells were not different between PF and GVHD-like skin lesions (Figure 3B). Moreover, the number of FoxP3^+^ Treg cells in the patient's thymoma at the time of recurrence was significantly lower compared to adult persistent thymus and indolent thymoma without associated autoimmune syndromes (Figure 3C).

### Peripheral Blood T Cell Response Against Cutaneous Autoantigen

By ELISpot assay, the peripheral blood T cell response of the patient against different cutaneous autoantigens, i.e., Dsg1, Dsg3, BP180, and BP230, was studied at different time points as recently described (21) (more detailed information is given in the Supplementary Materials).

Tetanus toxoid served as a control protein. Due to the strong immunosuppressive therapy, it was impossible to systematically study peripheral blood T cell responses against distinct cutaneous autoantigens (Supplementary Figure 1). Except for the initial treatment phase of PF with rituximab, Th1 and Th2 responses against the recall antigen, i.e., tetanus toxoid, were visible. After thymectomy, distinct Th1 and Th2 responses against Dsg3 and BP180 became apparent.

## Discussion

The association of thymoma, MG and PF is uncommon and is only rarely described in the literature (12). PF, MG, and thymoma can manifest as part of multiple autoimmune syndrome type 1, which consists of at least three autoimmune diseases including polymyositis, autoimmune thyroid disease, and giant cell myocarditis (18, 24). There have been sporadic cases of co-existing MG and PF which were not linked to thymoma (7, 8, 25). The etiopathogenesis of the simultaneous occurrence of MG, PF, and thymoma is only poorly understood. It has been proposed that a defective negative selection of autoreactive T-cells in neoplastic thymus leads to a loss of tolerance associated with autoantibody production against epithelial cells in Hassall's corpuscles and myeloid cells. These autoantibodies may then cross-react with homologous antigens of the epidermis and striate muscle, respectively (12). This contention is supported by the finding that the number of FoxP3^+^ Treg cells in thymomas is inversely correlated with the occurrence of associated autoimmune disorders, i.e., impaired Treg cell function favors the onset of autoimmune disorders (26, 27).

Tsuchisaka et al. reported on a patient with PF associated with thymoma which expressed higher levels of Dsg3, Dsc2, Dsc3, and autoimmune regulator (AIRE) but not Dsg1 compared to normal adult thymus, indolent thymomas, and thymic carcinoma. These findings suggest that lack of AIRE-induced expression of Dsg1 lead to an autoimmune response against this epidermal adhesion protein (28).

Our patient was diagnosed with PF based on the characteristic skin lesions and presence of anti-Dsg1 IgG antibodies. In addition, serum IgG against Dsc1 was also initially detected, and was likely linked to impaired desmosomal function (29). The patient did not fulfill all the criteria for PNP based on; (i) a lack of mucosal involvement, (ii) negative IIF on urinary bladder, and (iii) the absence of serum IgG antibodies against plakins, which are major autoantigens of PNP (6, 30).

Anti-laminin 332 antibodies are pathogenic autoantibodies in anti-laminin 332 mucous membrane pemphigoid, a distinct subepidermal autoimmune bullous disorder showing predominant mucosal lesions. However, the pathogenic role of anti-laminin 332 antibodies detected in the present case is unclear, as the patient neither presented subepithelial blisters on the skin nor on the mucous membranes. Due to the profound impaired immunological tolerance status, the presence of IgG autoantibodies against laminin 332 is likely the consequence of epitope spreading (31).

Although considered as a separate entity based on its characteristic spectrum of pathology, there is not a clear-cut distinction between thymoma associated multiorgan autoimmunity (TAMA) and PAMS. Accordingly, (i) thymomas are the second most frequent neoplasms associated with PAMS, following Castleman's disease (2); (ii) among the various cutaneous presentations, which also include pemphigus-like, BP-like or multiforme lesions, several cases of PAMS resembling cutaneous GVHD have been reported (2); (iii) involvement of liver and colon in PAMS has been also reported (2). As for thymoma, both PAMS and TAMA are thought to occur as a result of incomplete deletion of self-reactive T cells in the tumors (32). A decreased number of Treg cells in neoplastic thymus is believed to be also involved (33). In line with previous studies, we also observed a reduction of Treg cells in our patient's thymoma compared to adult thymus and indolent thymoma. The observed clinical spectrum, ranging from pemphigus-like to lichenoid lesions, likely depends on the T-cellular profile of the cutaneous immune response, either humoral (Th2), and later T cell-mediated (Th1).

IgG antibodies against envoplakin and periplakin are characteristic serological markers of PAMS, while anti-Dsg1 and anti-Dsg3 IgG antibodies are rarely seen (34–36). Conversely, anti-Dsc1-3 IgG antibodies are seen in almost 60% of PAMS cases, as the presence of anti-Dsc1 IgG in the present case. Although unusual, the detection of initial epidermal cell surface staining and, at a later time point, linear staining at the BMZ is a classic feature of PAMS. The presence of both patterns has been considered as a criteria for the diagnosis of PAMS (2, 37), while systematic studies on the prevalence of IgG antibodies against epidermal or BMZ antigens in TAMA are lacking. In addition, one case of cutaneous thymoma associated GVHD-like erythroderma was reported to show IgG deposits on the surface of epidermal keratinocytes by DIF (38).

In our case, even though IIF failed to show the presence of IgG autoantibodies against epidermal keratinocytes, DIF taken from perilesional skin at two different clinical stages showed initially the presence of IgG and C3 deposits on the surface of epidermal keratinocytes (Figure 2A) and, later on, the presence of linear IgG and C3 deposits at the BMZ (Figure 2B), which was supported by the detection of anti-Dsg1, anti-Dsc1, and anti-laminin 332 IgG antibodies. It could be argued that Dsg1, Dsc1, and/or laminin 332 function as antigenic targets and triggers for the activation of autoreactive T cells driving GVHD-like erythroderma (39). This contention is supported by a recent study of our group which suggested that lichenoid dermatoses such as LP harbor a T-cellular response against epidermal (Dsg3) or BMZ (BP180) autoantigens commonly associated with autoimmune blistering diseases (23). As recently suggested by Amber, it should be discussed if PNP should be classified as a subtype of PV or if PNP is just a variant of PAMS, a large group of clinically variable disorders which are all linked the occurrence of various neoplasias. We also tend to believe that PNP, as TAMA, should be considered as a variant of PAMS, but this requires a broader consensus (3). In conclusion, the present study proposes an immunologic conversion from an initial Th2-driven to a Th1-type dominated cutaneous immune response triggered by the recurrence of the thymoma (Figures 1, 3A). This shift to a Th1 response was paralleled by the complete resolution of the initial Th2 driven autoimmune disorders, PF and MG (Figure 1), and by the disappearance of serum anti-Dsg1/Dsc1 IgG antibodies (Table 1).

Of note, a study by Fujisao and Tsuda (40) described a patient with thymoma-associated pure red aplasia in which thymectomy resulted in an increased ratio of peripheral blood Th1/Th2 cells leading to significant amelioration of anemia (40). In 2015, Garrwan et al. reported on a patient with thymoma and associated proteinuria due to minimal change glomerulonephritis, in which antitumoral therapy with belinostat, cisplatin, doxorubicin, and cyclophosphamide resulted in a complete resolution of the tumor, reduction of proteinuria and increase in the Th1/Th2 ratio (41). Further accumulation of cases is required to better understand how thymectomy modulates thymoma-associated autoimmune syndromes via specific T-cell signatures and impacts on the course of these associated paraneoplastic syndromes.

## Data Availability

The datasets generated for this study are available on request to the corresponding author.

## Ethics Statement

In accordance with the declaration of Helsinki, written consent for the publication of this case report, and the accompanying images was obtained from the patient.

## Author Contributions

FS, RM, RP, and TS wrote the manuscript, performed the experiments, analyzed data, and designed figures. RE and MH designed and supervised the study. TH and NI provided help for extensive serological diagnostic. BT, AK, DD, and JP helped during clinical follow-ups and helped during manuscripts writing. RP, TS, RE, MH, and TH revised the study. All authors revised and finally approved the study.

### Conflict of Interest Statement

The authors declare that the research was conducted in the absence of any commercial or financial relationships that could be construed as a potential conflict of interest.

## Supplementary Material

Supplementary Figure 1 Analysis of peripheral blood T cell subset responses against cutaneous autoantigens during the disease course. ELISpot analysis of IL-10^+^, IFN-γ^+^, IL-17A^+^, and IL-5^+^ peripheral blood T cells against tetanus toxoid (recall antigen), bullous pemphigoid (BP) 180, BP230, desmoglein (Dsg) 1, and Dsg3. Numbers of autoreactive T cells were determined at the initial stage (pemphigus foliaceus), at thymoma recurrence, after thymectomy and at resolution of the lichenoid eruption.
