# Supplementary material for: Thymoma-Associated Paraneoplastic Autoimmune Multiorgan Syndrome—From Pemphigus to Lichenoid Dermatitis
Source: Front Immunol. 2019 Jun 21;10:1413. doi: 10.3389/fimmu.2019.01413 (PMC6598597; doi:10.3389/fimmu.2019.01413)
Supplement: Supplementary file 1 [file Data_Sheet_1.docx]

**Supplementary Materials**

**Autoantibody profile in the patient with thymoma-associated autoimmune syndrome**

IgG autoantibodies were analyzed in plasma samples from the patient by ELISA, according to the manufacturers’ instructions. The following ELISA assays were used: human Dsg1, Dsg3, BP180, BP230 (Euroimmun, Lübeck, Germany), and collagen VII (MBL, Nagoya, Japan). Autoantibodies profiling against different connective tissues and organs was analyzed by Biochip Mosaic (Euroimmun, Lübeck, Germany).

**Immunohistochemical analysis of the T cell infiltrate in skin lesions and thymoma tissue**

Staining of paraffin-embedded skin sections was performed with the automated IHC stainer BOND-MAX (Leica, Wetzlar, Germany) and Autostainer Plus automated immunostaining device (Dako, Hamburg, Germany) as recently described (1). The following primary antibodies were used: mouse anti-human CD3, and CD4, (all from Novocastra, Leica, Wetzlar, Germany); rabbit anti-human IL- 17A and rabbit anti-human FoxP3 (both from Novus, Littleton, Colo); and rabbit anti-human T-bet and rabbit anti-human GATA-3 (both from Cell Signaling Technology, Danvers, M). Secondary antibodies used were as follows: Bond Polymer Refine Detection Kit (Leica; CD3 and CD4) biotinylated anti-rabbit IgG, and biotinylated anti-mouse IgG (Vector Laboratories, Burlingame, CA; IL-17A, FoxP3, T-bet, and GATA-3). Antibodies from Vector Laboratories were subsequently detected by using peroxidase- or alkaline phosphatase–labeled ABC systems (Dako, Glostrup, Denmark). Visualization was carried out with 3,39-diaminobenzide or Liquid Permanent Red staining (both from Dako) as chromogenes. The T-cell infiltrate of skin lesions was quantified based on microscopic image (Axiostar; Zeiss, Jena, Germany) in combination with Cell^D software (Soft Imaging System, Berlin, Germany) and free ImageJ software (imagej.nih.gov/ij/). FoxP3^+^, IL-17A^+^, CD3^+^T-bet^+^, and CD4^+^GATA-3^+^ T cells were counted. After generating a grid (ImageJ software; area per point, 50,000 square pixels), all stained T cells were counted in 2 squares (4 squares for CD3^+^T‑bet^+^ and CD4^+^GATA-3^+^ T cells) adjacent to the BMZ (ImageJ software, cell counter), and their proportion of all infiltrating cells was determined afterward. FoxP3^+^ cell staining of thymoma tissues was performed and counted in an analogous manner.

**ELISpot Assay**

ELISpot assays were performed, as recently described (1). IFN-γ–, IL-5–, and IL-17A–spots were detected according to the manufacturers’ instructions (Human IFNg-ELISpot, Human IL-5-ELISpot; Becton Dickinson; Human IL-17A ELISpot Ready-Set-Go, eBioscience, San Diego, USA, CA). PBMCs were seeded at 1-3 x 10^5^ cells per well on ELISpot plates, and developed plates were finally analyzed with the ELISpot plate reader A.EL.VIS (A.EL.VIS, Hannover, Germany). For data analysis, spots of non-stimulated controls (mean) were subtracted from spots (mean) of cultures with antigen (all in duplicate). One spot was added to all values to exclude values of 0 to form ratios of distinct cytokine-producing T-cell subsets.

Reference:

1. Schmidt T, Solimani F, Pollmann R, Stein R, Schmidt A, Stulberg I, et al. TH1/TH17 cell recognition of desmoglein 3 and bullous pemphigoid antigen 180 in patients with lichen planus. J Allergy Clin Immunol. 2018;142(2):669-72 e7.
