# Supplementary figures and images for: Thymoma-Associated Paraneoplastic Autoimmune Multiorgan Syndrome—From Pemphigus to Lichenoid Dermatitis
Source: Front Immunol. 2019 Jun 21;10:1413. doi: 10.3389/fimmu.2019.01413 (PMC6598597; doi:10.3389/fimmu.2019.01413)

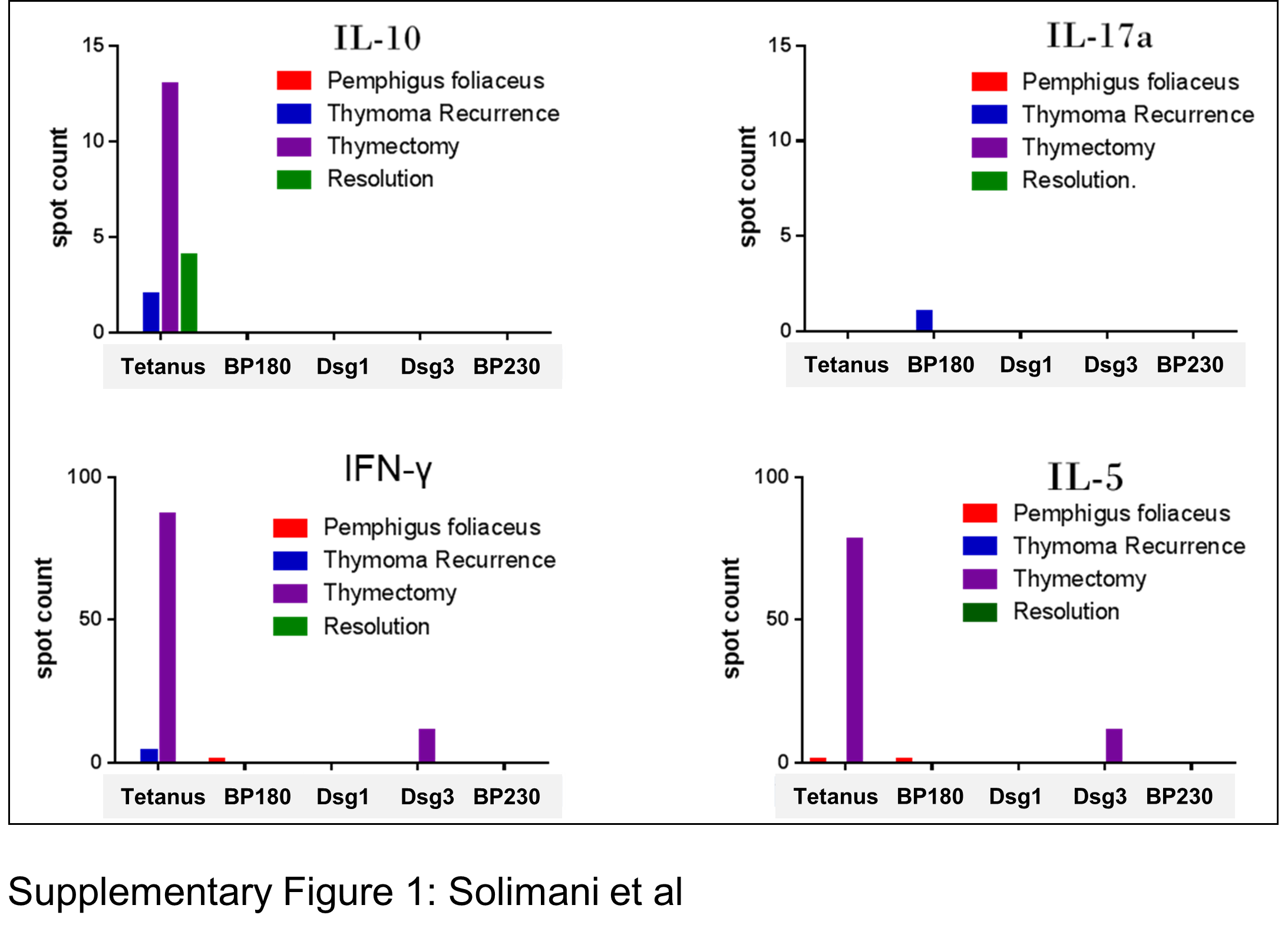

Supplement: Supplementary Figure 1 — Analysis of peripheral blood T cell subset responses against cutaneous autoantigens during the disease course. ELISpot analysis of IL-10+, IFN-γ+, IL-17A+, and IL-5+ peripheral blood T cells against tetanus toxoid (recall antigen), bullous pemphigoid (BP) 180, BP230, desmoglein (Dsg) 1, and Dsg3. Numbers of autoreactive T cells were determined at the initial stage (pemphigus foliaceus), at thymoma recurrence, after thymectomy and at resolution of the lichenoid eruption. [file Image_1.TIF]
